# Evidence for the association of the DAOA (G72) gene with schizophrenia and bipolar disorder but not for the association of the DAO gene with schizophrenia

**DOI:** 10.1186/1744-9081-5-28

**Published:** 2009-07-08

**Authors:** Nicholas J Bass, Susmita R Datta, Andrew McQuillin, Vinay Puri, Khalid Choudhury, Srinivasa Thirumalai, Jacob Lawrence, Digby Quested, Jonathan Pimm, David Curtis, Hugh MD Gurling

**Affiliations:** 1 Molecular Psychiatry Laboratory, Research Department of Mental Health Sciences, University College London Medical School, Windeyer Institute of Medical Sciences, 46 Cleveland Street, London, W1T 4JF, UK; 2 West Berkshire NHS Trust, 25 Erleigh Road, Reading, RG3 5LR, UK; 3 Department of Psychiatry, University of Oxford, Warneford Hospital, Headington, Oxford, UK; 4 Queen Mary College, University of London, 327 Mile End Rd London, E1 4NS, UK; 5 East London and City Mental Health Trust, Royal London Hospital, Whitechapel, London, E1 1BB, UK

## Abstract

**Background:**

Previous linkage and association studies have implicated the D-amino acid oxidase activator gene (DAOA)/G30 locus or neighbouring region of chromosome 13q33.2 in the genetic susceptibility to both schizophrenia and bipolar disorder. Four single nucleotide polymorphisms (SNPs) within the D-amino acid oxidase (DAO) gene located at 12q24.11 have also been found to show allelic association with schizophrenia.

**Methods:**

We used the case control method to test for genetic association with variants at these loci in a sample of 431 patients with schizophrenia, 303 patients with bipolar disorder and 442 ancestrally matched supernormal controls all selected from the UK population.

**Results:**

Ten SNPs spanning the DAOA locus were genotyped in these samples. In addition three SNPs were genotyped at the DAO locus in the schizophrenia sample. Allelic association was detected between the marker rs3918342 (M23), 3' to the DAOA gene and both schizophrenia (χ^2 ^= 5.824 p = 0.016) and bipolar disorder (χ^2 ^= 4.293 p = 0.038). A trend towards association with schizophrenia was observed for two other DAOA markers rs3916967 (M14, χ^2 ^= 3.675 p = 0.055) and rs1421292 (M24; χ^2 ^= 3.499 p = 0.062). A test of association between a three marker haplotype comprising of the SNPs rs778293 (M22), rs3918342 (M23) and rs1421292 (M24) and schizophrenia gave a global empirical significance of p = 0.015. No evidence was found to confirm the association of genetic markers at the DAO gene with schizophrenia.

**Conclusion:**

Our results provide some support for a role for DAOA in susceptibility to schizophrenia and bipolar disorder.

## Introduction

The lifetime risk of developing schizophrenia or bipolar disorder in the UK is 0.7% – 0.85% and 0.3% – 1.5% respectively. Family, twin and adoption studies have shown that there is a large genetic component to both disorders. Multiply affected families are common, and there is good evidence that the susceptibility genes for schizophrenia can be transmitted by obligate carriers who have an abnormal EEG, abnormal eye tracking and volumetric deficits on MRI but have no formal mental illness [1]. Replicated evidence from genetic linkage studies has confirmed that multiple chromosomal loci are involved in the heritability of both bipolar disorder and schizophrenia [2,3].

Evidence that a schizophrenia susceptibility locus maps to chromosome 13q has been reported by several groups [4-11], although not consistently demonstrated [2,12,13]. However such variability in the outcome of linkage studies is to be expected given genetic heterogeneity.

Several studies have also found linkage between bipolar disorder (BPD) and markers on chromosome 13q. The distribution of these linked markers would suggest at least two distinct regions of linkage: 13q11-13q21 and 13q22-13q32. The distal 13q22-13q32 region is more robustly supported by the linkage data. A meta-analysis of linkage scans in bipolar disorder found the strongest evidence for a susceptibility locus on 13q at 79 cM [14]. Shaw et al subsequently reported significant linkage with a lod score of 3.4 for markers mapping to this region [11]. In addition, there have been a number of independent reports which support linkage to the 13q22-13q32 region [15-18].

Chumakov and colleagues initially investigated the 13q34 region in schizophrenia using 191 SNPs spanning 5 Mb in French Canadian and Russian samples [19]. Six markers were found to be associated with schizophrenia in the French Canadian sample, two of which were also associated in the Russian sample. This region contains two overlapping genes, DAOA (G72) and G30, which are transcribed in opposing directions. The DAOA transcript was found to express a primate specific 153 amino acid protein that localises to the endoplasmic reticulum [19]. However, more recently it has been shown that the LG72 transcript codes for a mitochondrial protein [20]. No protein was identified for G30 in this study and it was postulated that because G30 is transcribed on the opposite strand it could act as a regulator for DAOA expression [19]. Expression studies of DAOA in post-mortem brain have found evidence for over expression in schizophrenic brains compared to controls, but not for G30 [21]. A correlation between DAOA and G30 expression was also found supporting the notion that G30 acts as a regulator of DAOA [21].

DAOA was found to interact directly with the enzyme D-amino acid oxidase (DAO) leading to activation of DAO [19]. However, this interaction could not be replicated by Kvajo et al [20]. The DAO gene has been localised to chromosome 12q. Four SNPs within the gene were also found to be associated with schizophrenia in the French Canadian but not in the Russian sample [19]. Logistic regression analysis demonstrated an interaction occurred between SNPs at the two loci [19]. DAO is known to oxidise D-serine, which is a powerful activator of the NMDA-type glutamate receptor [22]. It was found that levels of D-serine levels were higher in the serum of schizophrenic patients compared to normal controls [23]. Elevated levels of D-serine were also found in cerebrospinal fluid of drug naïve patients with schizophrenia [24].

Hattori et al first reported association of variants at the DAOA/G30 locus with bipolar disorder [25]. They examined 16 SNPs across a 157 kb region encompassing the DAOA/G30 locus in two family-based samples. Five SNPs showed individual association with BPD in the Clinical Neurogenetics (CNG) sample.

Subsequently a number of groups have attempted to replicate the associations between DAOA/G30 and schizophrenia, BPD, a psychosis phenotype, and panic disorder. A recent meta analysis of association findings for the DAOA/G30 locus published up until April 2007 found consistent evidence for association with markers at the locus with schizophrenia. [26]. rs947267 (M18 SCZ in Asians) and rs778293 (M22 SCZ in Asians) were associated with schizophrenia in Asian populations. rs1421292 (M24) was associated with schizophrenia in European populations.

A number of studies were not included in the meta analysis or have been published since April 2007. Hall et al, who studied US and Afrikaans trios with schizophrenia, found association between rs2391191 (M15) and schizophrenia in the Afrikaans sample [27]. Korostishevsky et al found suggestive evidence for association with schizophrenia at the DAOA locus in a Palestinian-Arab sample [28]. Fallin et al used an Israeli sample of 337 BPD trios and 274 schizophrenia trios. They showed overlapping suggestive evidence of association with DAOA/G30 for both disorders. They also found evidence for association of DAO with BPD [29]. Shin et al found association of rs947267 (M18) and rs778294 (M19) with schizophrenia in a Korean case control sample [30]. Shinkai et al reported association of rs746187 (M7) and rs947267 (M18) with schizophrenia in their case control sample. They also found over transmission of alleles to affected offspring with markers rs746187 (M7), rs3918342 (M23) and rs1421292 (M24) in their family based sample [31]. Corvin et al reported association with markers at both the DAO and DAOA loci in an Irish schizophrenia case control sample [32]. Association was detected with the DAOA markers rs3916965 (M12 p = 0.005) and rs2391191 (M15 p = 0.01); and with the DAO markers rs2111902 (M4 p = 0.02) and rs391834 (M5 p = 0.003). A logistic regression based interaction analysis demonstrated evidence for epistatic interaction between the DAOA marker rs3916965 (M21) and the DAO marker rs3918346 (M5), p = 0.008. Of note these were not the same markers found to interact in the Chumakov (2002) study [19]. Prata et al found no evidence for single marker association with bipolar disorder using markers at DAOA and DAO in a case control sample. They did however find some evidence for haplotype association at both loci [33].

A UK study looked at DAOA polymorphisms in both schizophrenia and bipolar samples [34]. Association was detected in the bipolar sample but not in the schizophrenia sample [34]. The authors went on to perform sub-analysis across traditional diagnostic boundaries. More significant associations were found when schizophrenia patients with a history of major mood disorder were added to the bipolar group. Shultze et al also attempted a phenotypic dissection of their bipolar sample. They concluded that the DAOA association was specifically linked to BPD patients with persecutory delusions [35]. The authors also reported an independent haplotypic, but not allelic replication, in a Polish sample[35].

However not all studies have supported the association of DAOA with schizophrenia [36,37]. Genome wide association studies of schizophrenia published to date [38-43] do not further implicate DAOA or DAO. Two SNPs, rs1981272 (p = 0.0315) and rs9519697 (p = 0.0445), in the DAOA gene region were found to be associated in the WTCCC bipolar disorder genome wide association study [44]. However there is sample overlap between this study and the studies by Williams et al [34] and Prata et al [33]. No SNPs in the DOAO or DAO regions were found to be associated with bipolar disorder in a meta analysis of genome wide association data that included amongst others the UCL samples described here and the WTCCC samples [45].

In this investigation we test for allelic and haplotypic association between markers at the DAOA locus in our schizophrenia and BPD samples. Additionally we test for association of markers within the DAO locus with schizophrenia.

## Materials and methods

### Sample

The case and control samples were recruited from London and South England and consist of 431 volunteers with schizophrenia, 303 volunteers with bipolar disorder and 443 control volunteers. For all groups subjects were included only if three out of four grandparents were of English, Irish, Welsh or Scottish descent.

"Volunteers were excluded if the other grandparent was of non Caucasian European ancestry (based on the EU countries before the 2004 enlargement). UK National Health Service multicentre and local research ethics approval was obtained and all subjects signed an approved consent form after reading an information sheet. All volunteers with schizophrenia or bipolar disorder were interviewed using the SADS-L [46]. All cases were selected on the basis of having a primary clinical diagnosis of schizophrenia or bipolar disorder and were then formally diagnosed if they achieved the probable level of the Research Diagnostic Criteria (RDC). Research subjects with brain damage prior to the onset of the disorder were excluded. The "supernormal" control subjects were also interviewed with the initial clinical screening questions of the SADS-L and selected on the basis of not having a family history of schizophrenia, alcoholism or bipolar disorder and for having no personal history of any RDC defined mental disorder.

Genomic DNA was extracted from frozen whole blood samples using a standard cell lysis, proteinase K digestion, phenol/chloroform, ethanol precipitation method. DNA samples were quantified using picogreen.

### Genotyping

Eleven SNPs at the DAOA locus were genotyped in the BPD, schizophrenia and control samples. Three SNPs were genotyped at the DAO locus in the schizophrenia and the control samples. We attempted to assay all four DAO SNPs described in the original Chumakov study, but we were unable to develop an assay for the SNP MDAO7 with the technology available to us [19]. SNPs rs1341402, rs2391191 (M15), rs1935062, rs947267 (M18), rs778294 (M19), rs954581, rs778293 (M22), rs3918342 (M23), rs1421292 (M24) as well as the 3 DAO SNPs (MDAO4, MDAO5 and MDAO6) were genotyped by KBiosciences (Hertfordshire, UK) which employs the KASPar SNP genotyping method. Duplicate DNA samples were incorporated on the microtitre plates for 17% of individuals in order to detect error and confirm the reproducibility of genotypes. SNPs rs3916965 (M12) and rs3916967 (M14), were typed using Pyrosequencing according to manufacturer's instructions (Biotage, Uppsala, Sweden).

### Statistical analysis

In order to confirm that the samples were genetically well matched, fifteen genetic markers at chromosomal loci, thought not to be involved in schizophrenia or bipolar disorder were genotyped in a subset of the sample (200 cases and 300 controls) and analyzed to detect genetic heterogeneity between cases and controls using Wright's Fst statistic with the program GDA [47]. No evidence for heterogeneity was observed.

In addition, a statistical test (CHECKHET) for detecting subjects with an atypical genetic background was employed [48].

Prior to association analysis, the program SCANGROUP (which is a subprogram of GENECOUNTING) was used to test whether there were differences in the haplotype frequencies between the 96 well microtitre plates. This may identify errors due to data entry or plate inversion. Once data was error checked, the data was analysed to confirm Hardy-Weinberg equilibrium (HWE). Markers with a lack of HWE in the control group were rejected and genotyping was repeated. Single marker allelic association analyses were performed using standard chi-squared tests. The genotypes were analysed for marker-to-marker linkage disequilibrium (LD) using GENECOUNTING/LDPAIRS. This computes D' and r^2 ^tests of LD and maximum likelihood estimates of haplotype frequencies from phase unknown case control data [49,50]. For the DAOA locus all data obtained from patients with schizophrena, BPD and controls were used to calculate LD. We genotyped markers within the DAO locus in our schizophrenia and control sample and used this data to calculate LD values.

Two and three marker haplotypic association was tested. The significance of any overall haplotype association was computed using a permutation test [49-51].

## Results

All the data collected in this study was found to be in Hardy Weinberg equilibrium and free from systematic errors.

Table 1 displays the allele frequencies and tests of association for the SNP markers genotyped at the DAOA locus schizophrenic patients, bipolar disorder patients and controls. The results for the DAO SNPs are found in table 2. Allelic association was detected with both diseases with marker rs3918342 (M23; schizophrenia χ^2 ^= 5.824 p = 0.016; bipolar disorder χ^2 ^= 4.293 p = 0.038; combined χ^2 ^= 7.044 p = 0.008). A trend toward association with schizophrenia was observed with rs3916967 (M14; χ^2 ^= 3.675 p = 0.055) and rs1421292 (M24; χ^2 ^= 3.499 p = 0.0615). For marker rs1421292 (M24) a nominally significant association was also detected in the combined analysis (χ^2 ^= 4.06 p = 0.044).

**Table 1:** Allele Frequencies and tests of association with schizophrenia and bipolar disorder on chromosome 13q33.2 at the DAOA locus.

Marker	Position on chromosome 13 (bp)	Allele Counts		χ^2 ^(1 d.f.)	p value
rs3916965 (M12)	104901361	C	T		
Controls		391	237		
Schizophrenia		309	223	2.101	0.147

rs3916967 (M14)	104915349	A	G		
Controls		397	235		
Schizophrenia		306	228	3.675	0.055

rs1341402	104913510	C	T		
Controls		138	488		
Bipolar		126	472	0.172	0.679

rs2391191 (M15)	104917447	A	G		
Controls		326	544		
Schizophrenia		305	473	0.521	0.470
Bipolar		246	360	1.468	0.226

rs1935062	104926137	A	C		
Controls		416	210	0.172	0.679
Bipolar		401	205	0.011	0.917

rs947267 (M18)	104937663	A	C		
Controls		502	368		
Schizophrenia		454	348	0.203	0.652
Bipolar		358	246	0.362	0.548

rs778294 (M19)	104940236	C	T		
Controls		639	237		
Schizophrenia		593	211	0.141	0.707
Bipolar		438	166	0.033	0.856

rs954581	104950267	C	T		
Controls		102	518		
Bipolar		123	479	3.221	0.073

rs778293 (M22)	104967200	A	G		
Controls		522	499		
Schizophrenia		338	293	0.93	0.335

rs3918342 (M23)	104983750	C	T		
Controls		444	412		
Schizophrenia		358	422	5.824	**0.016**
Bipolar		281	325	4.293	**0.038**
Combined		639	747	7.044	**0.008**

rs1421292 (M24)	104996236	A	T		
Controls		462	398		
Schizophrenia		388	402	3.499	0.062
Bipolar		301	305	2.337	0.126
Combined		689	707	4.059	**0.044**

**Table 2:** Allele Frequencies and tests of association with schizophrenia at the DAO locus on chromosome 12q24.11 in schizophrenia patients and controls.

Marker	Position on chromosome 12 (bp)	Allele Counts		χ^2 ^(1 d.f.)	p value
rs2111902 (MDAO-4)	107781213	A	C		
Controls		604	266		
Schizophrenia		522	226	0.025	0.875

rs3918346 (MDAO-5)	107784350	C	T		
Controls		655	221		
Schizophrenia		601	181	0.976	0.323

rs3741775 (MDAO-6)	107786069	T	G		
Controls		483	379		
Schizophrenia		418	354	0.587	0.444

**Figure 1:**
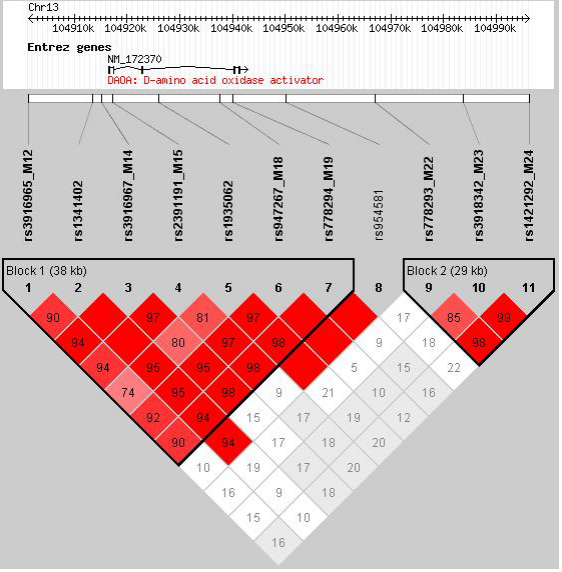
**Tests of linkage disequilibrium using D' between adjacent markers within the *DAOA/G30 *gene locus chromosome 13q33.2**.

Pair-wise linkage disequilibrium (LD) was calculated between all pairs of markers at the DAOA locus using GENECOUNTING for the combined data from the schizophrenia, BPD and control samples (see Figure 1). Markers rs3916965 (M12) to rs778294 (M19) formed an LD block defined with a solid spine of LD with a D' >0.8. Markers rs778293 (M22), rs3918342 (M23) and rs1421292 (M24) also formed a separate LD block. Tests of haplotypic association with schizophrenia with markers rs778293 (M22), rs3918342 (M23) and rs1421292 (M24) were positive (global permutation p = 0.018). The haplotype most likely to increase the risk of schizophrenia comprised alleles G, T, A with estimated frequencies of 1.8% in controls and 3.6% in cases of schizophrenia, the results of this analysis are shown in table 3. Haplotype analysis of data from the BPD sample did not show evidence for association.

**Table 3:** Estimated haplotype frequencies in controls and cases of schizophrenia with markers rs778293 (M22), rs3918342 (M23) and rs1421292 (M24) on chromosome 13q33.2 at the DAOA locus.

Alleles			Estimated frequencies	
M22	M23	M24	Controls	Schizophrenia

A	C	A	0.145	0.122

A	T	T	0.456	0.502

G	C	A	0.371	0.330

G	T	A	0.018	0.036

Association of rs3918342 (M23) using the following case definition was tested: schizophrenia with mood disorders, schizophrenia with mood disorders and BPD combined, BPD with persecutory delusions, and schizophrenia and BPD with persecutory delusions. Nominal association was found with the schizophrenia with mood disorders and BPD combined phenotype (χ^2 ^= 4.696 p = 0.030) and the schizophrenia and BPD with persecutory delusions phenotype (χ^2 ^= 3.968 p = 0.046).

**Figure 2:**
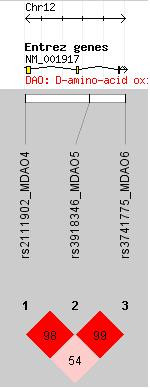
**Tests of linkage disequilibrium using D' between adjacent markers within the *DAO *gene locus chromosome 12q24.11**.

We did not find statistically significant association between the three markers at the DAO locus and schizophrenia (Table 2). D' values for DAO are displayed in Figure 2.

## Discussion

Allelic association was detected between one DAOA marker (rs3918342, M23) and schizophrenia in our sample. The T allele at rs3918342 (M23) was associated with schizophrenia in our study. This is the same allele that was found to be associated in the study of Chumakov et al [19]. However it should be stated that association between rs3918342 and schizophrenia was not found in the meta-analysis of DAOA studies [26]. A trend towards association with schizophrenia was found with the marker rs1421292 (M24). The T allele at rs1421292 (M24) was more common in schizophrenic patients than controls. This is the same allele that was found to be associated in the study of Chumakov et al [19] and the meta-analysis[26]. These findings were supported by haplotypic association.

A modest association of the marker rs3918342 (M23) was also found with bipolar disorder. This provides some support for the involvement of DAOA in the aetiology of bipolar disorder. It should however, also be noted that rs3918342 (M23) is located approximately 50 kb from the 3' end of DAOA and that the LD structure shown in figure 1 suggests that this SNP may have little relevance to unknown functional variants located within the gene.

Three markers were genotyped at the DAO locus in our schizophrenia sample. These did not provide evidence for allelic or haplotypic association with the disease.

In light of the previous findings with DAOA and DAO using the same markers and with the level of LD between markers no correction for multiple testing has been applied to the data presented because the appropriate method for correcting these analyses is not clear.

Our findings point to a role for DAOA in both schizophrenia and bipolar disorder. However it is evident that this locus can account for only a small proportion of the genetic susceptibility to these disorders.

## Competing interests

The authors declare that they have no competing interests.

## Authors' contributions

NJB, SRD, AM, VP, KC carried out the sample preparation and molecular genetic studies. NJB, SRD & AM drafted the manuscript. ST, JL, DQ, JP participated in the design of the study. DC oversaw the statistical analysis. HMDG conceived of the study, and participated in its design and coordination and helped to draft the manuscript. All authors read and approved the final manuscript.

## References

[B1] Gurling H, Papadimitriou GN, Mendlewicz J (1996). The Genetics of the schizophrenias. Genetics of Mental Disorders Part II: Clinical Issues, Balliere's Clinical Psychiatry, International Practice and Research.

[B2] Gurling HM, Kalsi G, Brynjolfson J, Sigmundsson T, Sherrington R, Mankoo BS, Read T, Murphy P, Blaveri E, McQuillin A, Petursson H, Curtis D (2001). Genomewide genetic linkage analysis confirms the presence of susceptibility loci for schizophrenia, on chromosomes 1q32.2, 5q33.2, and 8p21-22 and provides support for linkage to schizophrenia, on chromosomes 11q23.3-24 and 20q12.1-11.23. Am J Hum Genet.

[B3] Curtis D, Kalsi G, Brynjolfsson J, McInnis M, O'Neill J, Smyth C, Moloney E, Murphy P, McQuillin A, Petursson H, Gurling H (2003). Genome scan of pedigrees multiply affected with bipolar disorder provides further support for the presence of a susceptibility locus on chromosome 12q23-q24, and suggests the presence of additional loci on 1p and 1q. Psychiatr Genet.

[B4] Blouin JL, Dombroski BA, Nath SK, Lasseter VK, Wolyniec PS, Nestadt G, Thornquist M, Ullrich G, McGrath J, Kasch L, Lamacz M, Thomas MG, Gehrig C, Radhakrishna U, Snyder SE, Balk KG, Neufeld K, Swartz KL, DeMarchi N, Papadimitriou GN, Dikeos DG, Stefanis CN, Chakravarti A, Childs B, Pulver AE (1998). Schizophrenia susceptibility loci on chromosomes 13q32 and 8p21. Nat Genet.

[B5] Brzustowicz LM, Honer WG, Chow EW, Little D, Hogan J, Hodgkinson K, Bassett AS (1999). Linkage of familial schizophrenia to chromosome 13q32. Am J Hum Genet.

[B6] Camp NJ, Neuhausen SL, Tiobech J, Polloi A, Coon H, Myles-Worsley M (2001). Genomewide multipoint linkage analysis of seven extended Palauan pedigrees with schizophrenia, by a Markov-chain Monte Carlo method. Am J Hum Genet.

[B7] Faraone SV, Skol AD, Tsuang DW, Bingham S, Young KA, Prabhudesai S, Haverstock SL, Mena F, Menon AS, Bisset D, Pepple J, Sautter F, Baldwin C, Weiss D, Collins J, Keith T, Boehnke M, Tsuang MT, Schellenberg GD (2002). Linkage of chromosome 13q32 to schizophrenia in a large veterans affairs cooperative study sample. Am J Med Genet.

[B8] Levinson DF, Holmans P, Straub RE, Owen MJ, Wildenauer DB, Gejman PV, Pulver AE, Laurent C, Kendler KS, Walsh D, Norton N, Williams NM, Schwab SG, Lerer B, Mowry BJ, Sanders AR, Antonarakis SE, Blouin JL, DeLeuze JF, Mallet J (2000). Multicenter linkage study of schizophrenia candidate regions on chromosomes 5q, 6q, 10p, and 13q: schizophrenia linkage collaborative group III. Am J Hum Genet.

[B9] Lin MW, Curtis D, Williams N, Arranz M, Nanko S, Collier D, McGuffin P, Murray R, Owen M, Gill M (1995). Suggestive evidence for linkage of schizophrenia to markers on chromosome 13q14.1-q32. Psychiatr Genet.

[B10] Mulle JG, McDonough JA, Chowdari KV, Nimgaonkar V, Chakravarti A (2005). Evidence for linkage to chromosome 13q32 in an independent sample of schizophrenia families. Mol Psychiatry.

[B11] Shaw SH, Mroczkowski-Parker Z, Shekhtman T, Alexander M, Remick RA, Sadovnick AD, McElroy SL, Keck PE, Kelsoe JR (2003). Linkage of a bipolar disorder susceptibility locus to human chromosome 13q32 in a new pedigree series. Mol Psychiatry.

[B12] DeLisi LE, Shaw S, Crow TJ, Shields G, Smith AB, Larach VW, Wellman N, Loftus J, Nathankumar B, Razi K, Kushner M, Stewart J, Vita A, Comazzi M, Sherrington R (2000). Lack of evidence for linkage to chromosomes 13 and 8 for schizophrenia and schizoaffective disorder. Am J Med Genet.

[B13] Williams NM, Rees MI, Holmans P, Norton N, Cardno AG, Jones LA, Murphy KC, Sanders RD, McCarthy G, Gray MY, Fenton I, McGuffin P, Owen MJ (1999). A two-stage genome scan for schizophrenia susceptibility genes in 196 affected sibling pairs. Hum Mol Genet.

[B14] Badner JA, Gershon ES (2002). Meta-analysis of whole-genome linkage scans of bipolar disorder and schizophrenia. Mol Psychiatry.

[B15] Stine OC, McMahon FJ, Chen L, Xu J, Meyers DA, MacKinnon DF, Simpson S, McInnis MG, Rice JP, Goate A, Reich T, Edenberg HJ, Foroud T, Nurnberger JI, Detera-Wadleigh SD, Goldin LR, Guroff J, Gershon ES, Blehar MC, DePaulo JR (1997). Initial genome screen for bipolar disorder in the NIMH genetics initiative pedigrees: chromosomes 2, 11, 13, 14, and X. Am J Med Genet.

[B16] Potash JB, Zandi PP, Willour VL, Lan TH, Huo Y, Avramopoulos D, Shugart YY, MacKinnon DF, Simpson SG, McMahon FJ, DePaulo JR, McInnis MG (2003). Suggestive linkage to chromosomal regions 13q31 and 22q12 in families with psychotic bipolar disorder. Am J Psychiatry.

[B17] Liu J, Juo SH, Dewan A, Grunn A, Tong X, Brito M, Park N, Loth JE, Kanyas K, Lerer B, Endicott J, Penchaszadeh G, Knowles JA, Ott J, Gilliam TC, Baron M (2003). Evidence for a putative bipolar disorder locus on 2p13-16 and other potential loci on 4q31, 7q34, 8q13, 9q31, 10q21-24, 13q32, 14q21 and 17q11-12. Mol Psychiatry.

[B18] Cheng R, Juo SH, Loth JE, Nee J, Iossifov I, Blumenthal R, Sharpe L, Kanyas K, Lerer B, Lilliston B, Smith M, Trautman K, Gilliam TC, Endicott J, Baron M (2006). Genome-wide linkage scan in a large bipolar disorder sample from the National Institute of Mental Health genetics initiative suggests putative loci for bipolar disorder, psychosis, suicide, and panic disorder. Mol Psychiatry.

[B19] Chumakov I, Blumenfeld M, Guerassimenko O, Cavarec L, Palicio M, Abderrahim H, Bougueleret L, Barry C, Tanaka H, La Rosa P, Puech A, Tahri N, Cohen-Akenine A, Delabrosse S, Lissarrague S, Picard FP, Maurice K, Essioux L, Millasseau P, Grel P, Debailleul V, Simon AM, Caterina D, Dufaure I, Malekzadeh K, Belova M, Luan JJ, Bouillot M, Sambucy JL, Primas G, Saumier M, Boubkiri N, Martin-Saumier S, Nasroune M, Peixoto H, Delaye A, Pinchot V, Bastucci M, Guillou S, Chevillon M, Sainz-Fuertes R, Meguenni S, Aurich-Costa J, Cherif D, Gimalac A, Van Duijn C, Gauvreau D, Ouellette G, Fortier I, Raelson J, Sherbatich T, Riazanskaia N, Rogaev E, Raeymaekers P, Aerssens J, Konings F, Luyten W, Macciardi F, Sham PC, Straub RE, Weinberger DR, Cohen N, Cohen D (2002). Genetic and physiological data implicating the new human gene G72 and the gene for D-amino acid oxidase in schizophrenia. Proc Natl Acad Sci USA.

[B20] Kvajo M, Dhilla A, Swor DE, Karayiorgou M, Gogos JA (2008). Evidence implicating the candidate schizophrenia/bipolar disorder susceptibility gene G72 in mitochondrial function. Mol Psychiatry.

[B21] Korostishevsky M, Kaganovich M, Cholostoy A, Ashkenazi M, Ratner Y, Dahary D, Bernstein J, Bening-Abu-Shach U, Ben-Asher E, Lancet D, Ritsner M, Navon R (2004). Is the G72/G30 locus associated with schizophrenia? single nucleotide polymorphisms, haplotypes, and gene expression analysis. Biol Psychiatry.

[B22] Mothet JP, Parent AT, Wolosker H, Brady RO, Linden DJ, Ferris CD, Rogawski MA, Snyder SH (2000). D-serine is an endogenous ligand for the glycine site of the N-methyl-D-aspartate receptor. Proc Natl Acad Sci USA.

[B23] Hashimoto K, Fukushima T, Shimizu E, Komatsu N, Watanabe H, Shinoda N, Nakazato M, Kumakiri C, Okada S, Hasegawa H, Imai K, Iyo M (2003). Decreased serum levels of D-serine in patients with schizophrenia: evidence in support of the N-methyl-D-aspartate receptor hypofunction hypothesis of schizophrenia. Arch Gen Psychiatry.

[B24] Hashimoto K, Engberg G, Shimizu E, Nordin C, Lindstrom LH, Iyo M (2005). Reduced D-serine to total serine ratio in the cerebrospinal fluid of drug naive schizophrenic patients. Prog Neuropsychopharmacol Biol Psychiatry.

[B25] Hattori E, Liu C, Badner JA, Bonner TI, Christian SL, Maheshwari M, Detera-Wadleigh SD, Gibbs RA, Gershon ES (2003). Polymorphisms at the G72/G30 gene locus, on 13q33, are associated with bipolar disorder in two independent pedigree series. Am J Hum Genet.

[B26] Shi J, Badner JA, Gershon ES, Liu C (2008). Allelic association of G72/G30 with schizophrenia and bipolar disorder: a comprehensive meta-analysis. Schizophr Res.

[B27] Hall D, Gogos JA, Karayiorgou M (2004). The contribution of three strong candidate schizophrenia susceptibility genes in demographically distinct populations. Genes Brain Behav.

[B28] Korostishevsky M, Kremer I, Kaganovich M, Cholostoy A, Murad I, Muhaheed M, Bannoura I, Rietschel M, Dobrusin M, Bening-Abu-Shach U, Belmaker RH, Maier W, Ebstein RP, Navon R (2006). Transmission disequilibrium and haplotype analyses of the G72/G30 locus: suggestive linkage to schizophrenia in Palestinian Arabs living in the North of Israel. Am J Med Genet B Neuropsychiatr Genet.

[B29] Fallin MD, Lasseter VK, Avramopoulos D, Nicodemus KK, Wolyniec PS, McGrath JA, Steel G, Nestadt G, Liang KY, Huganir RL, Valle D, Pulver AE (2005). Bipolar I disorder and schizophrenia: a 440-single-nucleotide polymorphism screen of 64 candidate genes among Ashkenazi Jewish case-parent trios. Am J Hum Genet.

[B30] Shin HD, Park BL, Kim EM, Lee SO, Cheong HS, Lee CH, Kim SG, Sohn JW, Park CS, Kim JW, Kim BH, Kim IY, Choi IG, Woo SI (2007). Association analysis of G72/G30 polymorphisms with schizophrenia in the Korean population. Schizophr Res.

[B31] Shinkai T, De Luca V, Hwang R, Muller DJ, Lanktree M, Zai G, Shaikh S, Wong G, Sicard T, Potapova N, Trakalo J, King N, Matsumoto C, Hori H, Wong AH, Ohmori O, Macciardi F, Nakamura J, Kennedy JL (2007). Association analyses of the DAOA/G30 and D-amino-acid oxidase genes in schizophrenia: further evidence for a role in schizophrenia. Neuromolecular Med.

[B32] Corvin A, McGhee KA, Murphy K, Donohoe G, Nangle JM, Schwaiger S, Kenny N, Clarke S, Meagher D, Quinn J, Scully P, Baldwin P, Browne D, Walsh C, Waddington JL, Morris DW, Gill M (2007). Evidence for association and epistasis at the DAOA/G30 and D-amino acid oxidase loci in an Irish schizophrenia sample. Am J Med Genet B Neuropsychiatr Genet.

[B33] Prata D, Breen G, Osborne S, Munro J, St Clair D, Collier D (2008). Association of DAO and G72(DAOA)/G30 genes with bipolar affective disorder. Am J Med Genet B Neuropsychiatr Genet.

[B34] Williams NM, Green EK, Macgregor S, Dwyer S, Norton N, Williams H, Raybould R, Grozeva D, Hamshere M, Zammit S, Jones L, Cardno A, Kirov G, Jones I, O'Donovan MC, Owen MJ, Craddock N (2006). Variation at the DAOA/G30 locus influences susceptibility to major mood episodes but not psychosis in schizophrenia and bipolar disorder. Arch Gen Psychiatry.

[B35] Schulze TG, Ohlraun S, Czerski PM, Schumacher J, Kassem L, Deschner M, Gross M, Tullius M, Heidmann V, Kovalenko S, Jamra RA, Becker T, Leszczynska-Rodziewicz A, Hauser J, Illig T, Klopp N, Wellek S, Cichon S, Henn FA, McMahon FJ, Maier W, Propping P, Nothen MM, Rietschel M (2005). Genotype-phenotype studies in bipolar disorder showing association between the DAOA/G30 locus and persecutory delusions: a first step toward a molecular genetic classification of psychiatric phenotypes. Am J Psychiatry.

[B36] Mulle JG, Chowdari KV, Nimgaonkar V, Chakravarti A (2005). No evidence for association to the G72/G30 locus in an independent sample of schizophrenia families. Mol Psychiatry.

[B37] Goldberg TE, Straub RE, Callicott JH, Hariri A, Mattay VS, Bigelow L, Coppola R, Egan MF, Weinberger DR (2006). The G72/G30 Gene Complex and Cognitive Abnormalities in Schizophrenia. Neuropsychopharmacology.

[B38] Lencz T, Morgan TV, Athanasiou M, Dain B, Reed CR, Kane JM, Kucherlapati R, Malhotra AK (2007). Converging evidence for a pseudoautosomal cytokine receptor gene locus in schizophrenia. Mol Psychiatry.

[B39] Kirov G, Zaharieva I, Georgieva L, Moskvina V, Nikolov I, Cichon S, Hillmer A, Toncheva D, Owen MJ, O'Donovan MC (2008). A genome-wide association study in 574 schizophrenia trios using DNA pooling. Mol Psychiatry.

[B40] O'Donovan MC, Craddock N, Norton N, Williams H, Peirce T, Moskvina V, Nikolov I, Hamshere M, Carroll L, Georgieva L, Dwyer S, Holmans P, Marchini JL, Spencer CC, Howie B, Leung HT, Hartmann AM, Moller HJ, Morris DW, Shi Y, Feng G, Hoffmann P, Propping P, Vasilescu C, Maier W, Rietschel M, Zammit S, Schumacher J, Quinn EM, Schulze TG, Williams NM, Giegling I, Iwata N, Ikeda M, Darvasi A, Shifman S, He L, Duan J, Sanders AR, Levinson DF, Gejman PV, Cichon S, Nothen MM, Gill M, Corvin A, Rujescu D, Kirov G, Owen MJ, Buccola NG, Mowry BJ, Freedman R, Amin F, Black DW, Silverman JM, Byerley WF, Cloninger CR (2008). Identification of loci associated with schizophrenia by genome-wide association and follow-up. Nat Genet.

[B41] Shifman S, Johannesson M, Bronstein M, Chen SX, Collier DA, Craddock NJ, Kendler KS, Li T, O'Donovan M, O'Neill FA, Owen MJ, Walsh D, Weinberger DR, Sun C, Flint J, Darvasi A (2008). Genome-wide association identifies a common variant in the reelin gene that increases the risk of schizophrenia only in women. PLoS Genet.

[B42] Sullivan PF, Lin D, Tzeng JY, Oord E van den, Perkins D, Stroup TS, Wagner M, Lee S, Wright FA, Zou F, Liu W, Downing AM, Lieberman J, Close SL (2008). Genomewide association for schizophrenia in the CATIE study: results of stage 1. Mol Psychiatry.

[B43] Need AC, Ge D, Weale ME, Maia J, Feng S, Heinzen EL, Shianna KV, Yoon W, Kasperaviciute D, Gennarelli M, Strittmatter WJ, Bonvicini C, Rossi G, Jayathilake K, Cola PA, McEvoy JP, Keefe RS, Fisher EM, St Jean PL, Giegling I, Hartmann AM, Moller HJ, Ruppert A, Fraser G, Crombie C, Middleton LT, St Clair D, Roses AD, Muglia P, Francks C, Rujescu D, Meltzer HY, Goldstein DB (2009). A genome-wide investigation of SNPs and CNVs in schizophrenia. PLoS Genet.

[B44] WTCCC (2007). Genome-wide association study of 14,000 cases of seven common diseases and 3,000 shared controls. Nature.

[B45] Ferreira MA, O'Donovan MC, Meng YA, Jones IR, Ruderfer DM, Jones L, Fan J, Kirov G, Perlis RH, Green EK, Smoller JW, Grozeva D, Stone J, Nikolov I, Chambert K, Hamshere ML, Nimgaonkar VL, Moskvina V, Thase ME, Caesar S, Sachs GS, Franklin J, Gordon-Smith K, Ardlie KG, Gabriel SB, Fraser C, Blumenstiel B, Defelice M, Breen G, Gill M, Morris DW, Elkin A, Muir WJ, McGhee KA, Williamson R, MacIntyre DJ, MacLean AW, St CD, Robinson M, Van Beck M, Pereira AC, Kandaswamy R, McQuillin A, Collier DA, Bass NJ, Young AH, Lawrence J, Ferrier IN, Anjorin A, Farmer A, Curtis D, Scolnick EM, McGuffin P, Daly MJ, Corvin AP, Holmans PA, Blackwood DH, Gurling HM, Owen MJ, Purcell SM, Sklar P, Craddock N (2008). Collaborative genome-wide association analysis supports a role for ANK3 and CACNA1C in bipolar disorder. Nat Genet.

[B46] Spitzer R, Endicott J (1977). The schedule for affective disorders and schizophrenia, lifetime version.

[B47] Lewis PO, Zaykin D (2001). Genetic Data Analysis: Computer program for the analysis of allelic data. Book Genetic Data Analysis: Computer program for the analysis of allelic data (Editor ed ^eds), 10 edition City: Free program distributed by the authors over the internet from.

[B48] Curtis D, North BV, Gurling HM, Blaveri E, Sham PC (2002). A quick and simple method for detecting subjects with abnormal genetic background in case-control samples. Ann Hum Genet.

[B49] Curtis D, Knight J, Sham PC (2005). Program report: GENECOUNTING support programs. Ann Hum Genet.

[B50] Zhao JH, Lissarrague S, Essioux L, Sham PC (2002). GENECOUNTING: haplotype analysis with missing genotypes. Bioinformatics.

[B51] Zhao JH, Curtis D, Sham PC (2000). Model-free analysis and permutation tests for allelic associations. Hum Hered.

